# Supplementary material for: The impact of the COVID-19 pandemic on the use of restraint and seclusion interventions in Ontario emergency departments: A population-based study
Source: PLoS One. 2024 Apr 16;19(4):e0302164. doi: 10.1371/journal.pone.0302164 (PMC11020601; doi:10.1371/journal.pone.0302164)
Supplement: S1 Table — (PDF) [file pone.0302164.s001.pdf]

Table S1. Binary logistic regression model statistics.

Model	Step	Variable	B (*SE*)	Wald (*p*)	OR	95%CI
**Year**	**1**	**YEAR**	0.206 (.015)	184.96 (<.001)	1.23	(1.19 - 1.27)
		**CONSTANT**	-2.938 (.011)	72047.35 (<.001)	0.05	—
**Month * Year**	**1**	**YEAR**	0.196 (.052)	14.02 (<.001)	1.22	(1.10 - 1.35)
		Jan (ref)	—	*37.29 (<.001)*	—	—
		**Feb**	-0.072 (.055)	1.70 (.193)	0.93	(0.84 - 1.04)
		**Mar**	0.118 (.054)	4.71 (.030)	1.13	(1.01 - 1.25)
		**Apr**	-0.082 (.054)	2.29 (.130)	0.92	(0.83 - 1.03)
		**May**	-0.047 (.053)	0.77 (.379)	0.95	(0.86 - 1.06)
		**Jun**	0.046 (.053)	0.77 (.381)	1.05	(0.94 - 1.16)
		**Jul**	0.003 (.053)	0.00 (.962)	1.00	(0.90 - 1.11)
		**Aug**	0.095 (.052)	3.30 (.069)	1.10	(0.99 - 1.22)
		**Sep**	-0.056 (.054)	1.11 (.293)	0.95	(0.85 - 1.05)
		**Oct**	-0.057 (.054)	1.12 (.290)	0.95	(0.85 - 1.05)
		**Nov**	-0.096 (.055)	3.06 (.080)	0.91	(0.82 - 1.01)
		**Dec**	0.026 (.053)	0.23 (.630)	1.03	(0.92 - 1.14)
	**2**	***Jan * YEAR (ref)***	—	*109.48 (<.001)*	—	—
		**Feb * YEAR**	0.011 (.076)	0.02 (.882)	1.01	(0.87 - 1.18)
		**Mar * YEAR**	-0.219 (.075)	8.67 (.003)	0.80	(0.69 - 0.93)
		**Apr * YEAR**	0.408 (.075)	29.44 (<.001)	1.50	(1.30 - 1.74)
		**May * YEAR**	0.239 (.073)	10.71 (.001)	1.27	(1.10 - 1.47)
		**Jun * YEAR**	0.049 (.073)	0.46 (.499)	1.05	(0.91 - 1.21)
		**Jul * YEAR**	-0.041 (.073)	0.32 (.572)	0.96	(0.83 - 1.11)
		**Aug * YEAR**	-0.14 (.073)	3.72 (.054)	0.87	(0.75 - 1.00)
		**Sep * YEAR**	-0.106 (.075)	1.98 (.159)	0.90	(0.78 - 1.04)
		**Oct * YEAR**	-0.028 (.075)	0.14 (.705)	0.97	(0.84 - 1.13)
		**Nov * YEAR**	0.012 (.075)	0.03 (.875)	1.01	(0.87 - 1.17)
		**Dec * YEAR**	-0.011 (.074)	0.02 (.884)	0.99	(0.86 - 1.14)
		**CONSTANT**	-2.927 (.038)	5970.38 (<.001)	0.05	—
**MPDx * Year**	**1**	**YEAR**	0.163 (.038)	18.65 (<.001)	1.18	(1.09 - 1.27)
		EToH SUD (ref)	—	*3572.48 (<.001)*	—	—
		**OPI SUD**	-0.025 (.08)	0.09 (.759)	0.98	(0.83 - 1.14)
		**CBD SUD**	0.161 (.083)	3.75 (.053)	1.17	(1.00 - 1.38)
		**Poly SUD**	0.902 (.037)	579.44 (<.001)	2.47	(2.29 - 2.65)
		**Dementia**	1.114 (.121)	84.41 (<.001)	3.05	(2.40 - 3.87)
		**Psychosis**	1.153 (.035)	1112.46 (<.001)	3.17	(2.96 - 3.39)
		**Mood**	0.045 (.039)	1.36 (.244)	1.05	(0.97 - 1.13)
		**Anxiety**	-0.785 (.04)	391.22 (<.001)	0.46	(0.42 - 0.49)
		**Other MH**	0.324 (.072)	20.45 (<.001)	1.38	(1.20 - 1.59)

	**Personality**	0.551 (.066)	69.83 (<.001)	1.74	(1.53 - 1.98)
**2**	***EToH SUD * YEAR (ref)***	—	*30.35 (<.001)*	—	—
	**OPI SUD * YEAR**	-0.266 (.114)	5.41 (.020)	0.77	(0.61 - 0.96)
	**CBD SUD * YEAR**	0.116 (.11)	1.11 (.291)	1.12	(0.91 - 1.39)
	**Poly SUD * YEAR**	0.087 (.053)	2.75 (.097)	1.09	(0.98 - 1.21)
	**Dementia * YEAR**	0.141 (.16)	0.78 (.377)	1.15	(0.84 - 1.58)
	**Psychosis * YEAR**	-0.044 (.048)	0.81 (.368)	0.96	(0.87 - 1.05)
	**Mood * YEAR**	0.083 (.055)	2.26 (.133)	1.09	(0.98 - 1.21)
	**Anxiety * YEAR**	-0.133 (.057)	5.42 (.020)	0.88	(0.78 - 0.98)
	**Other MH * YEAR**	-0.038 (.101)	0.15 (.703)	0.96	(0.79 - 1.17)
	**Personality * YEAR**	0.045 (.089)	0.26 (.612)	1.05	(0.88 - 1.25)
	**CONSTANT**	-3.117 (.026)	13879.84 (<.001)	0.04	—
