# Supplementary material for: The impact of the COVID-19 pandemic on the use of restraint and seclusion interventions in Ontario emergency departments: A population-based study
Source: PLoS One. 2024 Apr 16;19(4):e0302164. doi: 10.1371/journal.pone.0302164 (PMC11020601; doi:10.1371/journal.pone.0302164)
Supplement: S2 Table — (PDF) [file pone.0302164.s002.pdf]

Table S2. Regression equation coefficients (B-weights) and variable values used to calculate R/S probability.

			INTERCEPT	YEAR		FACTOR		FACTOR * YEAR		STATISTICS		
VARIABLE	CATEGORY		B0	x	B1	z	B2	xz	B3	logOdds	Odds	*Probability*
YEAR		2019	-2.938	0	0.206	—	—	—	—	**-2.94**	**0.05**	***5.0%***
		2020	-2.938	1	0.206	—	—	—	—	**-2.73**	**0.07**	***6.1%***
MONTH	Jan	2019	-2.927	0	0.196	—	—	—	—	**-2.93**	**0.05**	***5.1%***
		2020	-2.927	1	0.196	—	—	—	—	**-2.73**	**0.07**	***6.1%***
	Feb	2019	-2.927	0	0.196	1	-0.072	0	0.011	**-3.00**	**0.05**	***4.7%***
		2020	-2.927	1	0.196	1	-0.072	1	0.011	**-2.79**	**0.06**	***5.8%***
	Mar	2019	-2.927	0	0.196	1	0.118	0	-0.219	**-2.81**	**0.06**	***5.7%***
		2020	-2.927	1	0.196	1	0.118	1	-0.219	**-2.83**	**0.06**	***5.6%***
	Apr	2019	-2.927	0	0.196	1	-0.082	0	0.408	**-3.01**	**0.05**	***4.7%***
		2020	-2.927	1	0.196	1	-0.082	1	0.408	**-2.41**	**0.09**	***8.3%***
	May	2019	-2.927	0	0.196	1	-0.047	0	0.239	**-2.97**	**0.05**	***4.9%***
		2020	-2.927	1	0.196	1	-0.047	1	0.239	**-2.54**	**0.08**	***7.3%***
	Jun	2019	-2.927	0	0.196	1	0.046	0	0.049	**-2.88**	**0.06**	***5.3%***
		2020	-2.927	1	0.196	1	0.046	1	0.049	**-2.64**	**0.07**	***6.7%***
	Jul	2019	-2.927	0	0.196	1	0.003	0	-0.041	**-2.92**	**0.05**	***5.1%***
		2020	-2.927	1	0.196	1	0.003	1	-0.041	**-2.77**	**0.06**	***5.9%***
	Aug	2019	-2.927	0	0.196	1	0.095	0	-0.140	**-2.83**	**0.06**	***5.6%***
		2020	-2.927	1	0.196	1	0.095	1	-0.140	**-2.78**	**0.06**	***5.9%***
	Sep	2019	-2.927	0	0.196	1	-0.056	0	-0.106	**-2.98**	**0.05**	***4.8%***
		2020	-2.927	1	0.196	1	-0.056	1	-0.106	**-2.89**	**0.06**	***5.3%***
	Oct	2019	-2.927	0	0.196	1	-0.057	0	-0.028	**-2.98**	**0.05**	***4.8%***
		2020	-2.927	1	0.196	1	-0.057	1	-0.028	**-2.82**	**0.06**	***5.6%***
	Nov	2019	-2.927	0	0.196	1	-0.096	0	0.012	**-3.02**	**0.05**	***4.6%***
		2020	-2.927	1	0.196	1	-0.096	1	0.012	**-2.82**	**0.06**	***5.7%***
	Dec	2019	-2.927	0	0.196	1	0.026	0	-0.011	**-2.90**	**0.05**	***5.2%***
		2020	-2.927	1	0.196	1	0.026	1	-0.011	**-2.72**	**0.07**	***6.2%***
MPDX	EToH SUD	2019	-3.117	0	0.163	—	—	—	—	**-3.12**	**0.04**	***4.2%***
		2020	-3.117	1	0.163	—	—	—	—	**-2.95**	**0.05**	***5.0%***
	OPI SUD	2019	-3.117	0	0.163	1	-0.025	0	-0.266	**-3.14**	**0.04**	***4.1%***
		2020	-3.117	1	0.163	1	-0.025	1	-0.266	**-2.25**	**0.11**	***9.6%***
	CBD SUD	2019	-3.117	0	0.163	1	0.161	0	0.116	**-2.96**	**0.05**	***4.9%***
		2020	-3.117	1	0.163	1	0.161	1	0.116	**-1.68**	**0.19**	***15.7%***
	Poly SUD	2019	-3.117	0	0.163	1	0.902	0	0.087	**-2.22**	**0.11**	***9.8%***
		2020	-3.117	1	0.163	1	0.902	1	0.087	**-0.97**	**0.38**	***27.6%***
	Dementia	2019	-3.117	0	0.163	1	1.114	0	0.141	**-2.00**	**0.13**	***11.9%***
		2020	-3.117	1	0.163	1	1.114	1	0.141	**-0.70**	**0.50**	***33.2%***
	Psychosis	2019	-3.117	0	0.163	1	1.153	0	-0.044	**-1.96**	**0.14**	***12.3%***
		2020	-3.117	1	0.163	1	1.153	1	-0.044	**-0.85**	**0.43**	***30.0%***
	Mood	2019	-3.117	0	0.163	1	0.045	0	0.083	**-3.07**	**0.05**	***4.4%***
		2020	-3.117	1	0.163	1	0.045	1	0.083	**-1.83**	**0.16**	***13.9%***
	Anxiety	2019	-3.117	0	0.163	1	-0.785	0	-0.133	**-3.90**	**0.02**	***2.0%***

	**2020**	-3.117	1	0.163	1	-0.785	1	-0.133	**-2.87**	**0.06**	***5.4%***
**Other MH**	**2019**	-3.117	0	0.163	1	0.324	0	-0.038	**-2.79**	**0.06**	***5.8%***
	**2020**	-3.117	1	0.163	1	0.324	1	-0.038	**-1.67**	**0.19**	***15.9%***
**Personality**	**2019**	-3.117	0	0.163	1	0.551	0	0.045	**-2.57**	**0.08**	***7.1%***
	**2020**	-3.117	1	0.163	1	0.551	1	0.045	**-1.36**	**0.26**	***20.5%***

Statistical formulas:

1. *logOdds = B0 + xB1 + zB2 + xzB3*
2. *Odds =* $e^{logOdds}$
3. *Probability = Odds / (1 + Odds)*
