# Supplementary material for: The impact of the COVID-19 pandemic on the use of restraint and seclusion interventions in Ontario emergency departments: A population-based study
Source: PLoS One. 2024 Apr 16;19(4):e0302164. doi: 10.1371/journal.pone.0302164 (PMC11020601; doi:10.1371/journal.pone.0302164)
Supplement: Attachment — Submitted filename: Response to Reviewers.pdf [file pone.0302164.s004.pdf]

Meghan Weissflog, PhD
Post-Doctoral Fellow
Waypoint Research Institute, Waypoint Centre for Mental Health Care
500 Church Street
Penetanguishene, Ontario
L9M 1G3, Canada
Phone: 1-705-549-3181 x 2542
Email: mweissflog@waypointcentre.ca

January 17, 2024

Subject: Rebuttal Letter/Response to Reviewers for Manuscript ID PONE-D-23-28304, titled "The impact of the COVID-19 pandemic on the use of restraint and seclusion interventions in Ontario emergency departments: A population-based study"

Dear Dr. Mishra,

I hope this letter finds you well. I am writing in response to the reviewers' comments on our manuscript titled "The impact of the COVID-19 pandemic on the use of restraint and seclusion interventions in Ontario emergency departments: A population-based study" (PONE-D-23-28304), which I submitted to PLOS ONE in September of 2023. I would like to express my gratitude to the reviewers for their constructive feedback, which has been helpful in revising our manuscript. Please find below detailed responses to each of the points raised by the reviewers.

1. Clear objective, and conclusion about interventions is help to understand the article more about study.

The reviewers correctly highlighted the importance of a clear objective and conclusion. In response to this, we have revised the manuscript to provide a more focused objective by elaborating on the fact that COVID-related changes in the use of restraint and seclusion interventions can be viewed as an indicator of the broader impact the COVID-19 pandemic had on patient experiences when seeking emergency care [pg. 5, lines 69-76; pg. 7, lines 119-123]. Additionally, we have added a "Future Directions" section to our conclusions to elaborate on avenues for potential intervention that may aid in preventing similar situations during future healthcare crises [pg. 19, lines 352-371].

2. Which kind of clinical feature present in MH/SU complaints?

We appreciate the reviewers' interest in better understanding the clinical features that accompanied presenting MH/SU complaints in the present sample. While the NACRS database does collect information regarding a patient's specific presenting complaints at the time of intake, this variable is optional in terms of NACRS data reporting guidelines and not consistently reported by participating facilities. As such, it was not possible to obtain robust data regarding specific clinical features that were present at the time of admission, so this variable was not included in the present study. Although the specific symptoms driving patients to seek emergency medical care are not known, attending physicians assign a main problem diagnosis,

based on the ICD-10 classification system, to indicate the underlying condition that is most likely responsible for the presenting complaint, taking into consideration a patient's current symptoms and medical history. Although the limitations of the current dataset prevent us from elaborating on this point, we agree that it is of great importance to further understand not only the impact of COVID-19 on specific symptoms experienced by individuals with MH/SU disorders but also which symptoms were most likely to result in the use of R/S interventions among these patients during the pandemic. To this end, we have added an additional paragraph to "Limitations" section addressing this issue [pg. 19-20, lines 370 -378].

3. What is impact of Clinical segregation of subjects on the results?

The reviewers have highlighted the importance of addressing the impact of clinical segregation on our results. When we examined the impact of COVID-19 on the use of seclusion alone, we observed a similar pattern of effects as that seen in the combined data (restraint and segregation), albeit to a lesser degree. Specifically, the probability of a seclusion event increased from pre-COVID levels by approximately 1% or less across registration month. The increase in probability for main problem diagnosis showed the same pattern as the original results in which diagnostic groups saw the greatest increases in seclusion probability, but to a smaller magnitude (~1-7% increase). These results suggest that the increases in probability seen in the combined data are primarily driven by restraint events more than seclusion events, which is consistent with the fact that there were three times more restraint events reported than seclusion events in 2019 and 2020. In the end, we chose to combine these two categories not only because they are both coercive methods of patient management and are subject to the same mandatory reporting guidelines but also because they generally show a similar pattern of effects when examined separately as they do when combined.

4. Graphical representation is important for better representation.

We agree with the reviewer's comment regarding the importance of graphical representation, and as such, we have added three new figures to the "Results" section to facilitate a better understanding of the data and ensure that readers can interpret the results more effectively [pg. 13-14, line 224, 227, 232; 234-248].

5. What is future prospect of the study?

To address the reviewers' comments regarding the future prospects of the study, we have expanded the discussion to include a "Future Directions" section as described above. This includes exploring additional factors associated with changes in the use of R/S interventions in response to the COVID pandemic and potential intervention strategies to prevent increased use of R/S methods during future healthcare crises [pgs. 19-21, lines 363-398].

6. Any comparative study in same region is important to understand the results.

The reviewers pointed out the importance of including comparable research conducted in the same region. To our knowledge, Martin et al. is the only other study that has looked at COVID-related changes in R/S intervention use in Ontario [described on pg. 5, lines 79-83]. The authors observed a substantial reduction in R/S intervention use, contrary to our findings in the present study. We think that the difference is because their sample was collected over a relatively short

period following the onset of COVID, and as such, is not necessarily reflective of the same conditions and systemic strain as was present in Ontario EDs.

While there does not appear to be any other comparable research on changes in R/S intervention use in EDs in the same geographical region, a number of studies from around the world have shown similar patterns of increased R/S use despite fewer people accessing care. This is consistent with the fact that COVID-19 was truly a pandemic, and like the virus itself, its impact on health care was not unique or confined to a specific geographical region. To this end, we have updated the "Introduction" section to include several newly published studies on this topic from around the world, the majority of which also found increases in R/S use [pg. 5, lines 83-88; pg. 6, lines 100-109].

7. What is public health model for other disease situations (epidemic-sporadic etc.)?

While we agree that it is important to consider the potential implications of our study's findings for other disease situations, the results of the present study are not as likely to be generalized to different disease scenarios, including epidemics and sporadic situations. While it is not impossible to see similar impacts on the use of R/S interventions given an epidemic of sufficient severity to overwhelm a regional health system, it is unlikely for endemic or sporadic disease events. This is because the driving factor behind the observed R/S increase during COVID was most likely the severe depletion of resources in the Canadian healthcare system, as illustrated by the fact that significant increases in R&S probability were confined to the first wave of the pandemic when the system was least prepared. Importantly, the observed increase in R&S intervention was not unique to COVID-19 or its related symptoms, but instead that its highly infectious nature and potential for severe outcomes resulted in a rapid increase in cases requiring substantial healthcare resources across the entire country (Canada) and more generally the world, in turn overwhelming an unprepared system. This was further exacerbated by the need for strict infection prevention protocols, which further strained already limited resources, both in terms of physical space and health care staff. It should be noted that public health restrictions intended to slow the spread of COVID-19 may have further exacerbated this issue by severely restricting patient access to non-emergency health care services and resources they may have previously accessed to address issues related to mental health and/or substance use concerns.

We believe that these revisions have significantly strengthened the manuscript, addressing the reviewers' concerns and improving the overall quality of the paper. We hope that these changes meet the expectations of the reviewers and the editorial board. Thank you for your time and consideration, as well as the opportunity to resubmit our revised manuscript for further evaluation.

Sincerely,

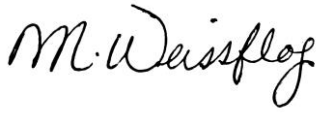

Meghan Weissflog, PhD
