# Supplementary material for: The impact of the COVID-19 pandemic on the use of restraint and seclusion interventions in Ontario emergency departments: A population-based study
Source: PLoS One. 2024 Apr 16;19(4):e0302164. doi: 10.1371/journal.pone.0302164 (PMC11020601; doi:10.1371/journal.pone.0302164)
Supplement: Attachment — Submitted filename: review.docx [file pone.0302164.s003.docx]

Meghan Weissflog and co-authors have reported an interesting data in the present study entitled “The impact of the COVID-19 pandemic on the use of restraint and seclusion interventions in Ontario emergency departments: A population-based study”. They examined the association between temporal and clinical factors and changes in the use of R/S interventions in Ontario EDs during the COVID pandemic. They observed that the visits to Ontario EDs decreased significantly for MH/SU concerns after the onset of the pandemic but despite this the number of visits where an R/S event occurred increased significantly. They have concluded that the pandemic was officially declared a public health emergency in Ontario on March 17, 2020, and the observed spike in the probability of R/S intervention use reflects the resulting impact on the public and healthcare.

Overall, this study has the potential to further the understanding of COVID-related changes in the presentation of MH/SU issues in ED facilities, as well as the ways in which the healthcare system responded to these changes during the pandemic and the data from this study can be used to improve the existing policies and procedures related to the use of R/S interventions to minimize the inherent risk to both patients and staff.
